# Metabolic Profile of Steers Subjected to Normal Feeding, Fasting, and Re-Feeding Conditions

**DOI:** 10.3390/vetsci7030095

**Published:** 2020-07-21

**Authors:** Enrico Lippi Ortolani, Celson Akio Maruta, Raimundo Alves Barrêto Júnior, Clara Satsuki Mori, Alexandre Coutinho Antonelli, Maria Claudia Araripe Sucupira, Antonio Humberto Hamad Minervino

**Affiliations:** 1 Department of Clinical Science, School of Veterinary Medicine and Animal Science, University of Sao Paulo, São Paulo-SP 05508-270, Brazil; celsomaruta@hotmail.com (C.A.M.); clarasat@usp.br (C.S.M.); msucupir@usp.br (M.C.A.S.); 2 Department of Animal Science, Federal University of Semiarid, Mossoró-RN 59625-900, Brazil; barreto@ufersa.edu.br; 3 School of Veterinary Medicine, Federal University of Sao Francisco Valley, Petrolina-PE 56304-205, Brazil; alexandre.antonelli@univasf.edu.br; 4 Laboratory od Animal Health, LARSANA, Federal University of Western Pará, UFOPA Santarém-PA 68040-255, Brazil

**Keywords:** metabolic profile, glucose, fasting, re-feeding, cattle

## Abstract

The effects of feeding, fasting, and re-feeding on the metabolic profile of growing cattle were studied. Blood and urine samples were obtained from 12 crossbred steers weighing approximately 300 kg during the following periods: 11 h of normal feeding (postprandial period), 48 consecutive hours of fasting, followed by 48 h of re-feeding. Compared with the postprandial period, fasting caused the following modifications: moderate hypoglycemia accompanied by remarkable lipolysis detected by the increase in plasma levels of free fatty acids (FFAs); absence of hepatic lipidosis, as there were no changes in aspartate aminotransferase activity or serum cholesterol levels; mild ketogenesis, confirmed by the slight increase of β-hydroxybutyrate (βHB); increased amino acid burn for energy production, verified by the increase in serum urea contents. There were strong positive correlations between the plasma levels of FFAs and βHB (*r* = 0.68; *p* < 0.001), fasting duration and FFA concentration (*r* = 0.92; *p* < 0.00001), and fasting duration and serum urea (*r* = 0.52; *p* < 0.001); there was a negative correlation between fasting duration and blood glucose (*r* = −0.52; *p* < 0.0001). During this same period, mild hypovolemia characterized by an increase in intravascular volume deficit was observed. The metabolic condition observed during fasting was completely reversed during re-feeding, except for the temporarily higher proteolysis.

## 1. Introduction

The metabolic profile is an important resource for laboratory evaluation of the nutritional and health statuses of cattle; it is susceptible to changes in the dietary management of animals. However, the intensity of dietary alterations and the time they require to produce effects on the metabolic profile has not been established, as studies report different outcomes [1,2,3]. Anorexia is a symptom of complex origin characterized by the complete absence of appetite and evidenced by the non-ingestion of food. The hypothalamus, especially through its centers of hunger and satiety, controls ingestion by modulating various hormones, blood concentrations of various metabolites, and the autonomic nervous system. The causes of anorexia are the most diverse, and they can generate changes in the blood metabolic profile, which is frequently used in veterinary clinical diagnosis [4,5,6].

During temporary or prolonged energy deficit, glucose production may be reduced, which can lead to hypoglycemia [7,8,9]. One of the most doubtful information is regarding the quantification of plasma glucose concentration in cattle, with the reference values used in Brazil retrieved from international literature [4,10,11]. However, the blood glucose concentrations used as reference values were obtained from lactating or pregnant dairy or beef cows and are lower compared to those in young ruminants [12,13,14], being approximately 20.6% lower than those measured by Kaneko et al. [11] and up to 64% lower than the values obtained by Payne and Payne [10]. This discrepancy calls the diagnosis of hypoglycemia in young cattle into question; under mild conditions, reduced blood glucose levels may still be within normal limits for cattle, which can lead to false interpretations and diagnostic errors.

Sucupira et al. [15] observed that intense and prolonged feed restriction resulted in marked hypoglycemia, however the glycemia cases were still within the range considered normal [11]. Studies that demonstrate the changes in the glucose concentrations during temporary fasting in young cattle and their relationships with those considered as reference values in the literature are lacking.

The simplest and most practical way to study the effects of complete food absence on the blood metabolic profile is through food fasting [9,16,17,18]. Although the effects of fasting on animal metabolism have been studied, they are not fully understood, especially in young and beef animals; changes in the blood metabolic profile caused by fasting, although they happen routinely, are reported as minimal or absent, and further studies are required. The effects of re-feeding, which can cause further changes in the metabolic profile, also need to be investigated.

Thus, this study aimed to evaluate the changes in the blood metabolic profile of beef steers that occur during the postprandial period, fasting, and re-feeding, with a focus on energy metabolism.

## 2. Material and Methods

This study was approved by the Ethics Commission on Animal Use of the School of Veterinary Medicine and Animal Science of the University of São Paulo (protocol no. 595/2004). Twelve 18-month-old healthy male crossbred steers with an initial mean weight of 300 kg were used for this study. The steers were kept in individual stalls with feed and water supply. During the adaptation period (30 days) and throughout the study, animals received water, mineral mixture (Fosbovi 20^®^, Tortuga, São Paulo, SP, Brazil), and a total mixed ration calculated by 2.7% of live weight (dry matter basis) of 70% of coast-cross grass hay and 30% of commercial concentrate, which was offered twice a day. Table 1 shows the chemical composition of the diet used.

The experiment consisted of three distinct and consecutive phases. All of the animals were submitted at the same time to treatments following a design for continuous flow response. In the first phase, called the postprandial period, blood and urine samples were obtained and evaluated before food supply and 1, 3, 5, 7, 9, and 11 h after morning feeding.

The second phase, called the fasting period, began on the morning of the day after the previous period and after the removal of all the food present in the feeder. Free access to water was maintained and mineralized salt was not offered during this period. The animals continued to fast for 48 h, and the blood and urine samples were obtained and evaluated before food removal and 12, 24, 36, and 48 h after food removal.

The third phase, called the re-feeding period, began at the end of the 48 h of fasting, and the experimental diet was offered at the same amounts and under the same conditions. The animals were followed for 48 h and the blood and urine samples were obtained and evaluated at baseline (the same sample from the 48 h fasting period was used) and 6, 12, 24, and 48 h after food supply.

Blood samples were obtained through venipuncture of the external jugular vein using vacuum collection tubes, with and without a coagulant, for biochemical evaluations. Whole blood samples were collected in 3-mL disposable plastic syringes containing sodium heparin for blood gas analyses [20].

The tubes containing blood without anticoagulant were kept at room temperature and were centrifuged after approximately 3 h of collection for 10 min at 250× *g* to obtain the serum, which was transferred to plastic tubes and stored at −20 °C until analyses. The tubes containing blood with sodium fluoride as an anticoagulant were immediately cooled and centrifuged after approximately 3 h of collection for 5 min at 250× *g* to obtain plasma, which was transferred to plastic tubes and stored at −20 °C.

Plasma was used to determine glucose, free fatty acids (FFA), and β-hydroxybutyrate (βHB) levels, and serum was used to determine cholesterol, urea, creatinine, aspartate aminotransferase (AST), total protein, and albumin. Biochemical analyses were performed using specific commercial diagnostic kits as follows: glucose (Sigma Diagnostics Inc., Livonia, MI, USA; Ref #315-100), FFA (Wako Chemical, Richmond, VA, USA; Ref#994-75409D), βHB (Sigma Diagnostics Inc.; Ref#310-A), cholesterol (Biosystems S.A., Barcelona, Spain; Ref#11805), AST (Biosystems; Ref#11830), urea (Bayer Diagnostic, New York, USA; Ref# T-01 1821-56), creatinine (Sigma Diagnostics Inc.; Ref#555), albumin, and protein (Sigma Diagnostics Inc.). The biochemical analyses were performed through an automatic biochemical analyzer (Liasic, AMS Alliance, Rome, Italy) according to protocols published elsewhere [21,22]. The serum globulin concentration was determined as the difference between total protein and albumin concentrations.

Urine samples were collected from the animals through a preputial massage that induced urination. Immediately after sampling, the urine samples were stored in a cooler at 4 °C and kept under refrigeration until they were transferred to the laboratory, then the Rothera test was performed. The Rothera test is a method of testing urine for the presence of acetone or acetoacetic acid (ketone bodies) and was determined using the Ketocheck diagnostic kit (Great States Animal Health, St. Joseph, MO, USA).

Plasma volume deficits (%) of protein, albumin, and globulin were calculated using the formula: plasma volume deficit of X (%) = (X1 (1−X2)/X2 (1−X1))−1 × 100, where X is the concentration of the biochemical variable (protein, albumin or globulin), X1 is the initial concentration, and X2 is the concentration at a subsequent time [11].

All variables were submitted to the Kolgomorov–Smirnov test to verify whether the distribution of the data was parametric. When the data had a non-Gaussian distribution, the variables were analyzed with the Mann–Whitney test and compared globally, considering the median for each period (postprandial, fasting, and re-feeding). For the evaluation of the influence of the day of collection (intragroup) and the treatment (intergroup), the data were initially submitted to the F test (ANOVA) and their means were compared using the Tukey multiple comparison test if they were significant.

Linear regression analysis and correlation coefficients were used to determine the relationship between two variables. The significance of the linear regression outcomes was evaluated using the F test. Differences were considered significant at *p* < 0.05. Correlations were considered high at *r* > 0.6, moderate at 0.3 < *r* < 0.6, and low at *r* < 0.3 [23].

## 3. Results

The Rothera test was negative in all the animals during all the periods evaluated. Table 2 presents the plasma glucose, FFA, and βHB concentrations. Blood glucose levels did not change during the postprandial period; however, the values obtained during the 36 and 48 h were lower than the initial values measured during the fasting period *(p* < 0.001). During the re-feeding period, the values were lower initially (*p* < 0.001); the glucose concentrations at 6 and 48 h of re-feeding were lower than at 12 h (*p <* 0.01). The FFA concentrations did not change during the postprandial period, but they continuously increased during fasting (*p* < 0.001); the opposite occurred during re-feeding *(p <* 0.05), except for at 24 h, which had similar values as at 12 and 18 h.

During the postprandial period, βHB concentrations were lower at baseline (*p* < 0.05) than at other times, except at 1 h. During fasting, the βHB concentrations at 12 h were lower than at other times *(p <* 0.001), except at 24 h. The βHB concentrations were higher at 48 h than 24 h (*p* < 0.01). During re-feeding, the baseline and 6-h concentrations of βHB were higher *(p* < 0.001).

Table 3 shows the mean blood creatinine, urea, cholesterol concentrations, and AST activity during each evaluation period. Lower creatinine concentrations were found at baseline and 48 h than at 6 and 12 h of the re-feeding period (*p* < 0.05). Lower serum urea concentrations were detected before food supply than at 3, 5, 7, and 9 h (*p* < 0.001) after feeding during the postprandial period. During the fasting period, higher concentrations of serum urea were observed at 24, 36, and 48 h than at baseline and 12 h (*p* < 0.01). During re-feeding, higher urea values were verified at 6 h *(p <* 0.05), followed by 12 h *(p <* 0.05), baseline *(p <* 0.01), and 24 and 48 h (*p* < 0.001). There were no differences between the cholesterol levels during the postprandial and fasting periods; the levels were higher initially than at 48 h (*p* < 0.05).

Table 4 shows the total protein, albumin, and globulin concentrations. There were no differences in serum protein levels during the postprandial period, but higher values were detected at 48 h in relation to 0 h during fasting (*p* < 0.001); during the re-feeding period, higher concentrations (*p* < 0.01) of serum protein were found before re-feeding in comparison to 12, 24, and 48 h after feeding. There were no differences in albumin concentrations during the postprandial period; higher concentrations were observed after 48 h of fasting than 0 h before food removal and after 12 h of fasting *(p* < 0.01); the same was observed during the re-feeding period, with higher concentrations of serum protein found at 0 h compared to 12, 24, and 48 h *(p <* 0.01).

There were no differences in serum globulin concentrations during the postprandial period; however, higher concentrations of globulins were observed at 48 h after fasting than at 0 and 12 h *(p <* 0.05). During re-feeding, albumin concentrations were higher at baseline (*p* < 0.01) than at 12, 24, and 48 h after feeding.

Table 5 presents the global assessments of the serum biochemical variables during the physiological periods (postprandial, fasting, and re-feeding). The global median for the serum protein was higher during fasting than the other periods (*p* < 0.01), while higher global medians of serum albumin were observed during fasting and re-feeding than during the postprandial period (*p* < 0.01). The global median of globulin was higher during fasting than during the postprandial and re-feeding periods (*p* < 0.01). There were no differences in serum creatinine concentrations during the postprandial and fasting periods; however, they were higher during re-feeding than during the postprandial period (*p* < 0.05). There were no differences between the global medians of urea during any of the periods. The global median of cholesterol was higher during fasting than during the postprandial period (*p* < 0.01). The AST activity global median was lower during the postprandial period (*p* < 0.05).

The overall median of the serum protein plasma volume deficit (PVD) was lower during fasting than during re-feeding (*p* < 0.001). There was no difference in albumin PVD, but the overall median of globulin PVD was lower during fasting than during re-feeding *(p <* 0.001).

Table 6 presents the results of the correlation analysis between variables during the fasting period. A high positive correlation was found between the plasma concentrations of FFAs and βHB. There were also positive correlations between the fasting duration and FFA concentration and the fasting duration and serum urea concentration; the fasting duration was negatively correlated with blood glucose.

## 4. Discussion

### 4.1. Energy Status: Fasting Contrasted with the Postprandial Period

The metabolic profile of the animals during the postprandial period was generally consistent with reports from Kaneko et al. [11]. As a typical example, the glycemic curve was characterized by a mean glucose concentration that was lower at the first postprandial hour than at the subsequent hours. This was to be expected because the absorption of propionate in the rumen causes a sudden release of insulin and a consequent moderate reduction in blood glucose [21,24,25].

Glycemia is considered a basic but important variable for the evaluation of energy status [10,26]. The blood glucose concentrations during the postprandial period of this study, considering two standard deviations around the mean (3.4 to 4.8 mmol/L; mean of 4.1 mmol/L), were well above the reported by Kaneko et al. [11] (2.5 to 4.2 mmol/L) and Payne and Payne [10] (2.0 to 3.0 mmol/L). These data were obtained in dairy, beef, lactating, or pregnant cows, which are known to have lower blood glucose concentrations than young cattle [12,13,14,15]. There should be two reference values for blood glucose—one each for adult and young cattle. This was also supported by Otto et al. [13] in Paraguay; they reported reference values of glucose (4.32 ± 0.72 mmol/L) in young beef cattle that were similar to those obtained in this experiment.

Blood glucose concentrations decreased significantly during the fasting period, which were more intense with a longer duration of food abstention. This was also observed by other researchers [25,27,28]. Despite the sharp decrease in blood glucose, the fasting animals maintained energy through indirect homeostasis; energy was generated through lipolysis and proteolysis. These were directly or indirectly proven in this study.

Fat is stored in the form of triacylglycerol. The lipase enzyme breaks this compound into glycerol and three molecules of free fatty acids. Under experimental conditions, it has been shown that fasting induces a decrease in insulin secretion and an increase in glucagon secretion in bovines. This hormonal condition is highly stimulating for the higher activity of lipase, as insulin is an inhibitor of this enzyme, while glucagon is a stimulator [28]. In this experiment, lipolysis was manifested by the substantial increase in serum levels of free fatty acids, which were higher with longer durations of fasting (*r* = 0.92; *p* < 0.0001).

Blood circulating glycerol and volatile fatty acids can generate glucose through gluconeogenesis in the liver and kidney. The first substrate enters the glycolytic pathway and converts into triose-phosphate, which in turn converts into glucose; this is a route widely used during fasting [29,30]. The second substrate enters the liver and oxidizes the mitochondria to generate ATP and acetyl-CoA, which combines with oxaloacetate and enters the Krebs cycle. Finally, glucose is formed through a pathway called gluconeogenesis [31].

When large amounts of free fatty acids are generated, oxaloacetate levels decrease and ketone bodies form; approximately 65% comprise βHB [31,32,33]. This compound can also be generated by butyrate produced in ruminal fermentation, which is absorbed into the rumen epithelium and transformed into βHB to be used as energy [21,24]. Normally, the βHB detected in plasma is generated from rumen fermentation. In the present study, the plasma concentrations of βHB during the postprandial period ranged from 0.35 mMol/L (minimum value before diet offer) to 0.67 mmol/L, which are similar to those obtained by Herdt et al. [34]. The concentration of butyrate in the portal vein of cattle that fasted for two days was 0, demonstrating that during fasting, ruminal butyrate did not contribute to the formation of blood βHB [30]. In this study, there was an increase in plasma βHB concentrations between 24 and 48 h of fasting. Although the final increase (0.57 mmol/L) of the βHB concentration was still within normal limits, βHB was predominantly influenced by the generation of ketone bodies, as a high positive correlation was observed (*r* = 0.68; *p* < 0.0001) between the plasma levels of FFA and βHB. This indicates that ketogenesis occurred, but it was not enough to cause ketosis, which can be caused in cattle when the concentration of βHB exceeds the plasma concentration of 1 mmol/L [31,34].

The ketone bodies produced during fasting were not sufficient to be detected in urine, which is usually characteristic of cases of ketosis. Rule et al. [28] subjected steers to fasting for eight days and demonstrated that plasma βHB increased during the second fasting day, reaching 0.6 mmol/L. This is similar to the findings of our study; the level was maintained until the 8th day of fasting. These results bring the classic but generic statements that ketonuria is common in ruminants submitted to fasting into question [4,11]. In particular, ketonuria can occur more frequently in fasting parturient cows; they have a characteristic hormonal profile and a high requirement for milk synthesis, which causes a notorious negative energy balance that favors lipolysis and generates much higher amounts of ketone bodies that exceed the renal threshold and allow its detection during the Rothera test [31,34]. Ketonuria was not observed in steers submitted to prolonged dietary energy deficiency [15].

The overall medians for cholesterol and AST during fasting showed a slight significant increase when compared to the postprandial period. Despite this significant increase, both variables were within the reference values [10]. In other words, the mobilization of free fatty acids for the liver did not cause lesions in hepatocytes and did not alter the esterification mechanisms of triglycerides; hepatic lipidosis was not induced.

The fasting cattle maintained energy through the mobilization and use of lipids and amino acids from their body reserves. In normal cattle, the serum urea concentrations are maximal between 3 and 9 h of the postprandial period, mainly due to ammonia absorption induced by the digestion of dietary nitrogen in the rumen, which is duly converted in the liver to urea in the cycle of the same name [35]. In this experiment, longer fasting duration was associated with higher serum urea concentrations (*r* = 0.52; *p* < 0.0001).This increase in urea may be attributed to a decreased peripheral uptake of amino acids and increased catabolism of labile protein reserves, resulting in an increased burn of amino acids to produce energy and urea synthesis, which was observed by Rule et al. [28] until the second day of fasting.

In this experiment, it was demonstrated that food fasting caused a moderate increase in plasma volume deficit rate (i.e., mild hypovolemia), evidenced by increases in albumin, total protein, and serum globulins of 6.1%, 9.3%, and 11.8%, respectively, after 48 h. Unlike in previous studies that detected high plasma volume deficit rates (around 35%), dehydration, and oliguria in cattle with ruminal lactic acidosis [6,36], we did not observe dehydration at the end of the fasting period. We observed a moderate hypovolemia, which was probably a consequence of the lower water intake caused by fasting, since a significant portion of the water consumption came from the water in the food composition. In a controlled experiment, Bond et al. [17] submitted 300 kg steers to food fasting and evaluated daily water intake; while during the control period the average water intake was 41 L, at the end of 48 h of fasting the consumption had reduced to only 8.5 L.

In summary, the 48 h food fasting caused moderate hypoglycemia in the animals, accompanied by increased lipolysis, which triggered a slight increase in the concentration of serum βHB of ketogenic origin, without the occurrence of ketonuria or hepatic lipidosis.

### 4.2. Energy Status: Re-Feeding Contrasted with the Fasting Period

On the one hand, if the fasting caused a significant change in energy metabolism in the short term, the re-feeding generated immediate recovery of the energy state. The results indicated that there was marked gluconeogenesis on the first day of re-feeding, accompanied by a drastic reduction in lipolysis and the generation of βHB by the ketogenic pathway. There was a transitional period characterized by a higher serum urea concentration during the first 6 h. Lomax and Baird [30], based on the amount of propionate, lactate, βHB, glycerol, and some amino acids in the hepatic artery, theorized that the main precursor substrate of glucose during re-feeding was lactate (54.4%), followed by propionate (16.9%) and some amino acids (4.4%). The high levels of serum urea obtained during the sixth hour of re-feeding may indicate that amino acids have a more important role in the formation of glucose.

The concentrations of FFAs decreased dramatically during re-feeding, and it would be logical to state that insulin secretion increased while glucagon secretion decreased, as this hormonal condition would lead to a reduction in lipolysis and also a decrease in amino acid burn [31]. Therefore, this situation may not have occurred, because at the beginning of re-feeding the plasma glucose was at its minimal concentration, which would stimulate the secretion of glucagon and growth hormone.

In summary, during re-feeding there was a rapid recovery of the mild hypoglycemia detected during fasting, with a marked drop in lipolysis and ketogenesis that was temporarily accompanied by increased amino acid burn for energy. The mild intravascular dehydration observed during fasting was resolved during re-feeding.

## 5. Conclusions

This study verified that steers submitted to 48 h of fasting presented with moderate hypoglycemia, an increase in amino acid burn for energy, marked lipolysis, and mild ketogenesis; they did not develop evident hepatic lipidosis. Re-feeding resulted in the rapid recovery of hypoglycemia and prompt interruption of lipolysis and ketogenesis. Fasting generated a slight increase in intravascular fluid deficit (moderate hypovolemia), which was promptly corrected during re-feeding.

## Figures and Tables

**Table 1:** Bromatological compositions of coast-cross hay, commercial concentrate, and global diet offered to cattle during the experimental period.

Composition	Hay	Concentrated	Global Diet
% DM (dry matter)	87.2	84.3	86.3
% CF (crude fiber)	31.2	6.4	23.7
% CP (crude protein)	7.1	17.6	10.3
% EE (ether extract)	1.8	5.1	2.8
% MM (mineral matter)	6.4	9.5	7.3
% NNFE * (non-nitrogen free extract)	53.6	61.4	55.9
% TDN (total digestible nutrients)	54.3	74.9	60.5
% NDF (neutral detergent soluble fiber)	76.2	27.0	61.4
% ADF (acid detergent soluble fiber)	38.1	12.4	30.4
CE (crude energy) ** (kcal/kg)	4224	4275	4240

* Calculated by the difference of DM, CP, MM, EE, and CF. ** CE = crude energy of the diets of the treatments using the formula CE = ((5.72 × %CP) + (9.5 × %EE) + (4.79 × %CF) + (4.03 × %NNFE)) × 10, extracted from [19].

**Table 2:** Mean values and standard deviations of plasma glucose concentrations (mmol/L), free fatty acids (μMol/L), and β-hydroxybutyrate (mMol/L) of cattle in the postprandial (PP), fasting (F), and re-feeding (RF) periods.

Times		Glucose		Free Fatty Acids		β-Hydroxybutyrate	
		(mmol/L)		(μmol/L)		(mmol/L)	
PP	0 h	4.1 ± 0.7	^a^	124 ± 50	^a^	0.45 ± 0.09	^b^
	1 h	3.9 ± 0.4	^a^	122 ± 44	^a^	0.49 ± 0.09	^ab^
	3 h	4.0 ± 0.5	^a^	120 ± 40	^a^	0.54 ± 0.09	^a^
	5 h	4.1 ± 0.5	^a^	108 ± 33	^a^	0.56 ± 0.12	^a^
	7 h	4.2 ± 0.5	^a^	111 ± 40	^a^	0.57 ± 0.10	^a^
	9 h	4.2 ± 0.5	^a^	114 ± 31	^a^	0.54 ± 0.09	^a^
	11 h	4.3 ± 0.5	^a^	109 ± 49	^a^	0.55 ± 0.07	^a^
F	0 h	4.5 ± 0.8	^a^	124 ± 59	^e^	0.54 ± 0.03	^ab^
	12 h	4.4 ± 0.7	^a^	317 ± 135	^d^	0.39 ± 0.06	^c^
	24 h	4.3 ± 0.7	^a^	622 ± 126	^c^	0.46 ± 0.08	^bc^
	36 h	3.8 ± 0.6	^b^	821 ± 113	^b^	0.51 ± 0.10	^ab^
	48 h	3.5 ± 0.3	^b^	1115 ± 272	^a^	0.57 ± 0.19	^a^
RF	0 h	3.5 ± 0.3	^c^	1115 ± 272	^a^	0.57 ± 0.19	^a^
	6 h	4.0 ± 0.3	^b^	509 ± 141	^b^	0.52 ± 0.13	^a^
	12 h	4.8 ± 0.4	^a^	245 ± 121	^c^	0.39 ± 0.13	^b^
	24 h	4.5 ± 0.3	^ab^	141 ± 48	^cd^	0.35 ± 0.10	^b^
	48 h	4.3 ± 0.2	^b^	91 ± 38	^d^	0.33 ± 0.07	^b^

Distinct letters (^a^, ^b^, ^c^, ^d^, ^e^, ^ab^, ^bc^) in the same column indicate significant differences within the same physiological period (*p* < 0.05).

**Table 3:** Mean values and standard deviations of serum creatinine, urea, cholesterol, and aspartate aminotransferase (AST) activity of cattle in the postprandial periods (PP), fasting (F), and re-feeding (RF).

Times		Creatinine		Urea		Cholesterol		AST	
		(μmol/L)		(mmol/L)		(mmol/L)		(U/L)	
PP	0 h	159 ± 21	^a^	3.74 ± 1.80	^b^	4.11 ± 0.92	^a^	30.6 ± 2.9	^a^
	1 h	159 ± 17	^a^	4.39 ± 1.76	^ab^	3.98 ± 0.75	^a^	31.5 ± 3.0	^a^
	3 h	162 ± 19	^a^	5.49 ± 1.92	^a^	4.00 ± 0.79	^a^	31.9 ± 3.5	^a^
	5 h	163 ± 22	^a^	6.00 ± 2.00	^a^	3.86 ± 0.68	^a^	31.5 ± 3.2	^a^
	7 h	162 ± 22	^a^	5.79 ± 1.99	^a^	3.85 ± 0.62	^a^	32.2 ± 4.2	^a^
	9 h	162 ± 20	^a^	5.41 ± 2.06	^a^	3.95 ± 0.70	^a^	31.6 ± 4.9	^a^
	11 h	161 ± 18	^a^	4.86 ± 2.05	^ab^	3.92 ± 0.72	^a^	35.1 ± 7.0	^a^
F	0 h	162 ± 17	^a^	3.35 ± 1.73	^b^	4.03 ± 0.76	^a^	35.4 ± 6.8	^a^
	12 h	160 ± 19	^a^	4.04 ± 1.34	^b^	4.11 ± 0.91	^a^	36.6 ± 6.5	^a^
	24 h	167 ± 24	^a^	5.48 ± 1.20	^a^	4.24 ± 0.85	^a^	35.8 ± 6.5	^a^
	36 h	168 ± 30	^a^	5.49 ± 1.23	^a^	4.20 ± 0.68	^a^	35.0 ± 5.2	^a^
	48 h	169 ± 33	^a^	5.59 ± 1.46	^a^	4.34 ± 0.77	^a^	34.5 ± 4.0	^a^
RF	0 h	169 ± 33	^b^	5.59 ± 1.46	^c^	4.34 ± 0.77	^a^	34.5 ± 4.0	^a^
	6 h	185 ± 38	^a^	8.30 ± 1.51	^a^	4.18 ± 0.64	^ab^	35.2 ± 4.7	^a^
	12 h	185 ± 37	^a^	6.97 ± 1.68	^b^	3.92 ± 0.64	^ab^	33.8 ± 4.8	^a^
	24 h	179 ± 36	^ab^	3.81 ± 1.84	^d^	3.93 ± 0.76	^ab^	33.7 ± 4.6	^a^
	48 h	165 ± 25	^b^	3.31 ± 1.46	^d^	3.85 ± 0.68	^b^	33.4 ± 5.9	^a^

Distinct letters (^a^, ^b^, ^ab^) in the same column indicate significant differences within the same physiological period (*p* < 0.05).

**Table 4:** Mean values and standard deviations of serum concentrations of total protein, albumin, and globulin of cattle in the postprandial periods (PP), fasting (F), and re-feeding (RF).

Times		Total Protein		Albumin		Globulin	
		(g/L)		(g/L)		(g/L)	
PP	0 h	68.0 ± 6.3	^a^	31.5 ± 1.7	^a^	36.5 ± 6.0	^a^
	1 h	68.6 ± 5.2	^a^	31.7 ± 1.3	^a^	37.0 ± 5.1	^a^
	3 h	68.1 ± 6.2	^a^	31.4 ± 1.7	^a^	36.7 ± 5.7	^a^
	5 h	67.3 ± 5.1	^a^	31.1 ± 1.4	^a^	36.1 ± 5.0	^a^
	7 h	67.9 ± 6.2	^a^	31.2 ± 1.4	^a^	36.8 ± 6.1	^a^
	9 h	69.2 ± 5.3	^a^	31.4 ± 1.5	^a^	37.8 ± 5.1	^a^
	11 h	67.2 ± 5.0	^a^	31.4 ± 1.4	^a^	35.8 ± 4.8	^a^
F	0 h	69.2 ± 5.1	^b^	31.5 ± 1.2	^b^	37.7 ± 4.8	^b^
	12 h	71.5 ± 4.9	^ab^	31.8 ± 1.7	^b^	39.6 ± 4.7	^ab^
	24 h	71.6 ± 6.5	^ab^	32.2 ± 1.7	^ab^	39.4 ± 6.1	^ab^
	36 h	70.9 ± 4.7	^ab^	32.5 ± 1.2	^ab^	38.3 ± 4.8	^ab^
	48 h	75.2 ± 5.8	^a^	33.4 ± 1.7	^a^	41.8 ± 5.5	^a^
RE	0 h	75.2 ± 5.8	^a^	33.4 ± 1.7	^a^	41.8 ± 5.5	^a^
	6 h	71.5 ± 4.5	^ab^	33.2 ± 1.2	^a^	38.3 ± 4.5	^ab^
	12 h	68.4 ± 5.3	^b^	31.9 ± 1.3	^b^	36.5 ± 5.2	^b^
	24 h	69.4 ± 2.6	^b^	32.3 ± 1.1	^ab^	37.1 ± 2.9	^b^
	48 h	67.3 ± 6.2	^b^	31.6 ± 2.2	^b^	35.6 ± 4.6	^b^

Distinct letters (^a^, ^b^, ^ab^) in the same column indicate significant differences within the same physiological period (*p* < 0.05).

**Table 5:** Overall evaluation of biochemical variables in different physiological periods (postprandial, fasting, and feedback). Median values of serum concentrations of total protein, globulin, creatinine, urea, cholesterol, aspartate aminotransferase (AST), and plasma volume deficit (PVD) calculated by protein, albumin, and globulin levels.

Variable	Physiological Period						*p*
	Postprandial		Fasting		Re-Feeding		
Total Protein (g/L)	68.9	^b^	73.0	^a^	70.0	^b^	<0.01
Globulin (g/L)	38.1	^b^	40.2	^a^	36.7	^b^	0.01
Creatinine (μmol/L)	160	^b^	166	^ab^	177	^a^	0.05
Urea (mmol/L)	4.81	^a^	5.15	^a^	5.98	^a^	0.52
Cholesterol (mmol/L)	3.7	^b^	4.0	^a^	3.9	^ab^	<0.01
AST (U/L)	31.8	^b^	35.4	^a^	33.9	^a^	<0.05
Pt DVP (%)	ND		−4.5	^b^	0.1	^a^	0.0005
Albumin DVP (%)	ND		−3.0	^a^	−2.7	^a^	0.45
DVP Globulin (%)	ND		−4.3	^b^	2.2	^a^	0.0001

ND: not available; PVD: plasma volume deficit; EN: total protein; AST: aspartate aminotransferase. Distinct letters (^a^, ^b^, ^ab^) in the same row indicate significant differences between physiological periods (*p* < 0.05).

**Table 6:** Correlation between blood variables and fasting duration during the fasting time.

Variable	Glucose	FFA	βHB	Urea
Fasting time (h)	*r* = −0.52; *p* < 0.0001	*r* = 0.92; *p* < 0.00001	*r* = 0.33; *p* > 0.05	*r* = 0.52; *p* < 0.001
Glucose		*r* = −0.23; *p* > 0.05	*r* = −0.25; *p* > 0.05	*r* = −0.12; *p* > 0.05
FFA			*r* = 0,68; *p* < 0.0001	*r* = 0.41; *p* > 0.05
βHB				*r* = 0.37; *p* > 0.05
